# Hypertension and Subclinical Vascular Damage After Allogeneic Hematopoietic Stem Cell Transplantation

**DOI:** 10.1016/j.jacadv.2026.103314

**Published:** 2026-09-22

**Authors:** Wejdan Bin Obaid, Mar Bellido, Arie M. van Roon, Anouschka Biswana, Fleurine Meppelink, Michaël V. Lukens, Hilde Kooistra, Goda Choi, Linde Morsink, Carin Hazenberg, Gerwin Huls, Joop D. Lefrandt

**Affiliations:** a Department of Hematology, University Medical Centre Groningen, University of Groningen, Groningen, the Netherlands; b University of Groningen, Groningen, the Netherlands; c Division of Vascular Medicine, Department of Internal Medicine, University Medical Centre Groningen, Groningen, the Netherlands; d Department of Laboratory Medicine, University Medical Centre Groningen, Groningen, the Netherlands

**Keywords:** ambulatory blood pressure monitoring, allogeneic hematopoietic stem cell transplantation, cardiovascular disease, cardiovascular risk factors, hypertension, subclinical vascular damage

## Abstract

**Background:**

Patients with hematological malignancies undergoing allogeneic hematopoietic stem cell transplantation (alloHSCT) face a higher risk of premature cardiovascular disease that may partly be explained by hypertension.

**Objectives:**

The authors studied the prevalence of hypertension in alloHSCT survivors at least 1 year post-transplant.

**Methods:**

Two hundred survivors were clinically screened for hypertension. Hypertension was defined according to established ambulatory blood pressure monitoring (ABPM) thresholds. Subclinical vascular damage was assessed through carotid intima-media thickness by ultrasonography of the carotid arteries, pulse wave velocity, and renal function.

**Results:**

Of the 200 patients, 168 (84%) completed the 24-hour ABPM. The median age was 57 years (IQR: 46.3-65.0; range: 18-75). Of the 168 patients, 90 patients (53.6%) had hypertension, of which 26 (15.5%) were newly diagnosed, and 56 (33.3%) were untreated. Hypertensive patients by ABPM tended to be older (median 58 [IQR: 49-67] vs 55.5 [IQR: 39.75-64] years; *P* = 0.060), had a higher intima-media thickness (0.84 ± 0.25 vs 0.76 ± 0.26 mm; *P* = 0.009), higher pulse wave velocity values (9.0 ± 2.3 vs 7.3 ± 1.6 m/s; *P* < 0.001), and lower mean estimated glomerular filtration rate (72.3 ± 18.7 vs 80.4 ± 22.5 mL/min/1.73 m^2^; *P* = 0.010) compared to normotensive patients. In the logistic regression model, both age in years (OR: 1.03; 95% CI: 1.01-1.05; *P* = 0.019) and family history of cardiovascular disease and/or premature death (OR: 2.28; 95% CI: 1.17-4.44; *P* = 0.016) were independently associated with hypertension.

**Conclusions:**

Hypertension is highly prevalent, underrecognized, and undertreated in alloHSCT survivors and is cross-sectionally associated with subclinical vascular and renal organ damage.

Allogeneic hematopoietic stem cell transplantation (alloHSCT) has been successfully used as a curative approach for a variety of hematological malignancies and nonmalignant diseases.^1^ Improvements in the alloHSCT procedure and advances in supportive clinical care before and after alloHSCT have led to an increase in the number of long-term survivors.^2^ Long-term survivors, however, suffer from associated late effects that contribute to significant morbidity and mortality after alloHSCT.^2^, ^3^, ^4^ In fact, mortality rates of survivors after alloHSCT are higher than the general population, resulting in a 30% decrease in their life expectancy.^3^^,^^5^ The main causes of mortality in long-term survivors are infections, malignancies (late recurrence or secondary cancer), death from cardiovascular (CV) events, and chronic graft-versus-host disease (GvHD).^2^^,^^5^^,^^6^

A previous study reported a 2-fold higher incidence rate of hypertension in patients treated with alloHSCT.^7^ Other studies show that this potentially modifiable cardiovascular risk factor tends to be overlooked in daily practice.^7^^,^^8^ Also, the modality that is used in the outpatient clinic to identify hypertension is suboptimal.^9^

Twenty-four-hour ambulatory blood pressure monitoring (ABPM) is considered the most accurate method for measuring blood pressure (BP). It reduces variability and has a superior predictive value for cardiovascular disease (CVD) compared to office-based measurements.^9^ Moreover, 24-hour ABPM enables the detection of white coat hypertension, defined as elevated BP readings in the clinical office settings despite having normal BP in the ABPM readings at home, thus preventing overtreatment.^9^^,^^10^

Differences in the night/day BP ratio are referred to as dipping status.^10^^,^^11^ The absence of a decline in BP at night is called “non-dipping,” and it is associated with inflammation and target organ damage (such as atherosclerosis and arterial stiffness) and increased risk for overt clinical damage (such as kidney injury and CVD), both in the general population and in the alloHSCT patients.^10^^,^^12^, ^13^, ^14^, ^15^ One proposed link between hypertension and the development of atherosclerosis is changes in the nocturnal BP dipping status and morning surge, which contribute to vascular damage increasing the carotid intima-media thickness (IMT), a marker of subclinical atherosclerosis.^12^^,^^16^

Furthermore, the specific risk factors remain complex and poorly understood.^7^^,^^8^ Different mechanisms have been proposed that contribute to the development of hypertension after alloHSCT.^2^^,^^5^ For instance, previous studies in pediatric populations suggested that treatment-related factors such as pretransplant treatment, conditioning regimens (including intensive chemotherapy and radiation), and immunosuppressants, such as calcineurin inhibitors (eg, cyclosporine and tacrolimus), after alloHSCT are associated with the development of hypertension in alloHSCT survivors.^2^^,^^5^^,^^7^^,^^8^

The primary aim of the study is to assess the prevalence of hypertension in patients treated with an alloHSCT, surviving at least 1 year after transplant, using 24-hour ABPM. The secondary aim is to assess patient-related and treatment-related factors associated with the presence of hypertension in alloHSCT survivors. Additionally, we aim to evaluate whether hypertension is related to subclinical vascular damage, as assessed by the IMT of the carotid arteries, pulse wave velocity (PWV), and renal function.

## Methods

### Ethical approval

All patients provided written informed consent for this study. Data collection and analysis of patients required no further approval according to Dutch law. The University Medical Center Groningen (UMCG) Medical Ethics Review Board (Ref: M25.350592; METc 2025/218; RR 22118) concluded that the study does not fall under the Dutch Medical Research Involving Human Subjects Act (WMO), the European Union Medical Device Regulation (MDR), or the European Union Clinical Trials Regulation (CTR); therefore, no full ethical review or formal approval was required.

### Study design and population

This prospective cross-sectional study was conducted at the UMCG, the tertiary academic center in the north of the Netherlands, and included 200 consecutive adult patients who were diagnosed with hematological malignancy and treated with an alloHSCT between January 1998 and December 2019 and survived at least 1 year after transplant. The study was designed to assess the prevalence and characteristics of hypertension in alloHSCT survivors, with the long-term goal of informing future cardiovascular screening strategies. Patients were invited for cardiovascular screening, irrespective of the time since transplantation.

Inclusion criteria were: ≥18 years of age, having received an alloHSCT for hematological malignancy, complete remission of the hematological malignancy at the time of vascular evaluation, having survived ≥1 year after transplant, and having given written informed consent for this study. Patients were followed post-transplant at least once a year at the Department of Hematology according to JACIE guidelines (documentation is available at http://www.jacie.org). CV evaluation was conducted at least once on all included patients between January 2018 and December 2021 at the Department of Internal Medicine. Baseline parameters were recorded at the date of transplant.

All eligible patients meeting the inclusion criteria were consecutively included in the study.

To reduce information bias, we used standardized and validated tools for cardiovascular and laboratory data collection, cross-checking retrospective information from patient records where possible, and addressed missing data through exclusion or appropriate statistical methods.

At the time of hematological follow-up, all relevant data were collected from medical records, including the status of the hematological disease before alloHSCT, its actual status, previous treatments, current medication, and complications after transplantation (acute and chronic GvHD and infections). Additional information about the transplant procedure was retrospectively retrieved from patients’ medical files including donor source, mismatches (sex, human leukocyte antigen, cytomegalovirus, Epstein-Barr virus, blood, and Rhesus factor), and type of conditioning.

Additional lab values were obtained during the CV evaluation at the follow-up, including hemoglobin (Hb), leucocytes, platelets, ferritin, glucose, HbA1c percentage, triglycerides, total cholesterol, low-density lipoprotein, high-density lipoprotein (HDL), C-reactive protein, serum creatinine, and estimated glomerular filtration rate (eGFR).

Traditional cardiovascular risk factors (CVRFs) were assessed from medical records and cardiovascular screening data, including family history of CVD or premature cardiovascular death in a first-degree relative (<55 years in males and <65 in females). Pretransplant CVRFs were extracted from patients’ files, including smoking status (categorized into never, ever but in the past, and present smokers), obesity (body mass index ≥30 kg/m^2^), history of diabetes mellitus type 1 or type 2 (fasting glucose >7 mmol/L or random plasma glucose >11.1 mmol/L or HbA1c >6.5% or current glucose-lowering treatment), history of hypertension (defined as office BP ≥140/90 mm Hg or use of antihypertensive medication), dyslipidemia (defined as fasting low-density lipoprotein cholesterol >4.0 mmol/L, HDL cholesterol <1.2 mmol/L (female) or HDL-cholesterol <1.0 mmol/L (male), triglycerides >4.0 mmol/L, or current lipid-lowering medication).^17^

### Endpoints

Hypertension was classified according to the European Society of Cardiology 2016 guidelines. Conventional office BP measurements were simultaneously obtained in both arms on the day of screening included the mean of 2 different measurements for each arm according to the American Heart Association 2017 recommendations using a clinically validated oscillometric BP monitoring system. Twenty-four-hour ABPM measurement was performed and analyzed according to the guidelines of the European Society of Hypertension 2013.^16^ The ambulatory readings were obtained at 30-minute intervals over 24 hours. Daytime ranged between 8:00 AM and 10:00 PM. Night-time ranged between 10:00 PM and 8:00 AM. Hypertension was defined as having a 24-hour average systolic blood pressure (SBP) >130 mm Hg or diastolic BP (DBP) >80 mm Hg, or a daytime SBP >135 mm Hg or DBP >85 mm Hg, or a night-time SBP >120 mm Hg or DBP >70 mm Hg. Office BP was defined as BP measured in the clinical setting using standardized conventional methods, with hypertension defined as SBP ≥140 mm Hg and/or DBP ≥90 mm Hg. Patients were classified at cardiovascular screening according to 4 complementary frameworks. First, based on medical records before ABPM, patients were categorized as having a previous clinical diagnosis of hypertension or no previous diagnosis of hypertension. Second, based on ABPM at screening, patients were categorized as normotensive or hypertensive according to predefined ambulatory BP thresholds. Third, treatment status among patients with hypertension was classified as untreated hypertension, treated but uncontrolled hypertension, or treated and controlled hypertension. Untreated hypertension was defined as hypertensive ABPM in the absence of antihypertensive medication. Treated but uncontrolled hypertension was defined as hypertensive ABPM despite antihypertensive treatment. Treated and controlled hypertension was defined as use of antihypertensive medication with ABPM values below the hypertension threshold. Fourth, BP phenotype was classified by comparing office BP and ABPM as masked hypertension (normal office BP with hypertensive ABPM) or white-coat hypertension (elevated office BP with nonhypertensive ABPM).

The dipping ratio was calculated by dividing night-time 24-hour SBP, 24-hour DBP, and 24-hour heart rate by their corresponding daytime values. A non-dipper BP pattern was defined as a night/day ratio difference <10%, a dipper BP as a night/day ratio difference between 10% and 20%, an extreme dipper as a night/day ratio difference >20%, and an inverted dipper as a night/day ratio difference <0%. A successful ABPM recording was defined as a monitoring duration of ≥22 hours with ≥80% valid readings. Recordings not meeting these criteria were excluded. Time-weighted averages were used, and laboratory quality-control procedures ensured minimal missing data.

The evaluation parameters after alloHSCT included CV events that occurred before and after transplant, markers of subclinical vascular damage, and markers of kidney damage. CV events were classified as cardiac events (myocardial infarction and/or angina pectoris); cerebrovascular events (transient ischemic attack or cerebrovascular accident); peripheral artery disease (abdominal aortic aneurysm and/or peripheral artery occlusive disease); and revascularization procedure (coronary bypass grafting and percutaneous coronary intervention).

Subclinical organ damage was evaluated based on the following variables: carotid IMT, PWV, and renal function markers (eGFR and serum creatinine). IMT was measured by ultrasound readings, as described earlier.^17^ The mean and max IMT of the far wall of 6 different segments in the left and right carotid arteries were used in the assessment (bulbus, common carotid artery, and internal carotid artery). The presence of plaque was assessed according to the Mannheim consensus statement, which defines plaque as “a focal structure that encroaches into the arterial lumen at least 0.5 mm or 50% of the surrounding IMT value or demonstrates a thickness of ≥1.5 mm as measured from the media-adventitia interface to the intima-media lumen interface.”^18^ The plaque score was calculated by summing all segments with the presence of at least 2 or more plaques.

### Statistical analysis

Descriptive statistics were calculated for demographic, transplant-related, and cardiovascular outcome variables and given as mean (SD), median (Q1-Q3), and number (percentage) where appropriate. Data distribution was assessed for normality using the Shapiro-Wilk test. Normally distributed variables were compared using 2-tailed Student’s *t*-tests, while non-normally distributed variables were analyzed using Mann-Whitney *U* or Kruskal-Wallis tests. Categorical variables were analyzed using Pearson’s chi-square or Fisher exact tests, depending on expected cell counts, and are presented as counts (N) and percentages (%). For comparison of non-normally distributed groups, values were logarithmically transformed and given as geometric means where appropriate.

Univariate analyses were conducted to examine the relationships between traditional CVRFs, treatment-related variables, and the primary outcome. Multivariable logistic regression models were used to examine factors independently associated with hypertension. The following variables were included age, sex, history of diabetes, history of dyslipidemia, history of smoking (ever), family history of CVD or cardiovascular events, conditioning regimen, total-body irradiation exposure (>8 Gy), human leukocyte antigen mismatch, acute GvHD, chronic GvHD, time since transplant was evaluated as a categorical variable (<5 years, 5-10 years, and >10 years), and use of immunosuppressants for 1 year or more. Variables were selected for inclusion in the multivariable model based on clinical relevance, prior literature, or a univariate analysis threshold of *P* < 0.10. Candidate variables were subsequently entered into a multivariable logistic regression model, and variables with the highest *P* value were sequentially removed using a manual backward stepwise selection procedure. The final multivariable model retained only age and family history, defined as the presence of CVD and/or premature cardiovascular death. ORs and 95% CIs are reported for the final model.

Laboratory and vascular outcomes were excluded from the regression model due to multicollinearity and interaction with CVRFs. All patient data were fully anonymized prior to analysis. Statistical analyses were performed using IBM-SPSS Statistics software version 26, with statistical significance set at *P* < 0.05.

## Results

### Baseline characteristics of the alloHSCT patients

The first 200 screened patients were included in the study and underwent cardiovascular evaluation after alloHSCT at the Department of Vascular Medicine in the UMCG between January 2018 and December 2021, after at least 1 year from alloHSCT at the Department of Hematology. Of the 200 screened patients, 168 (84%) had valid 24-hour ABPM recordings and were included in the final analysis (Figure 1). A total of 32 patients were excluded due to having <80% of the 24-hour measurement intervals completed. Patients were categorized based on ABPM (SBP 24 hours) into a normotensive by ABPM group (n = 78) and a hypertensive by ABPM group (n = 90). The study included 69 (41.1%) female and 99 (58.9%) male patients. The median age at hematologic diagnosis was 49 years (IQR: 37.3-57.0; range: 14-74) (Table 1), and the median age at cardiovascular screening was 57 years (IQR: 46.3-65.0; range: 18-75) (Table 2). median time from alloHSCT to cardiovascular screening was 5 years (IQR: 2-9; range: 1-23). Baseline pretransplant and transplant-related characteristics are presented in Table 1.

**Figure 1:**
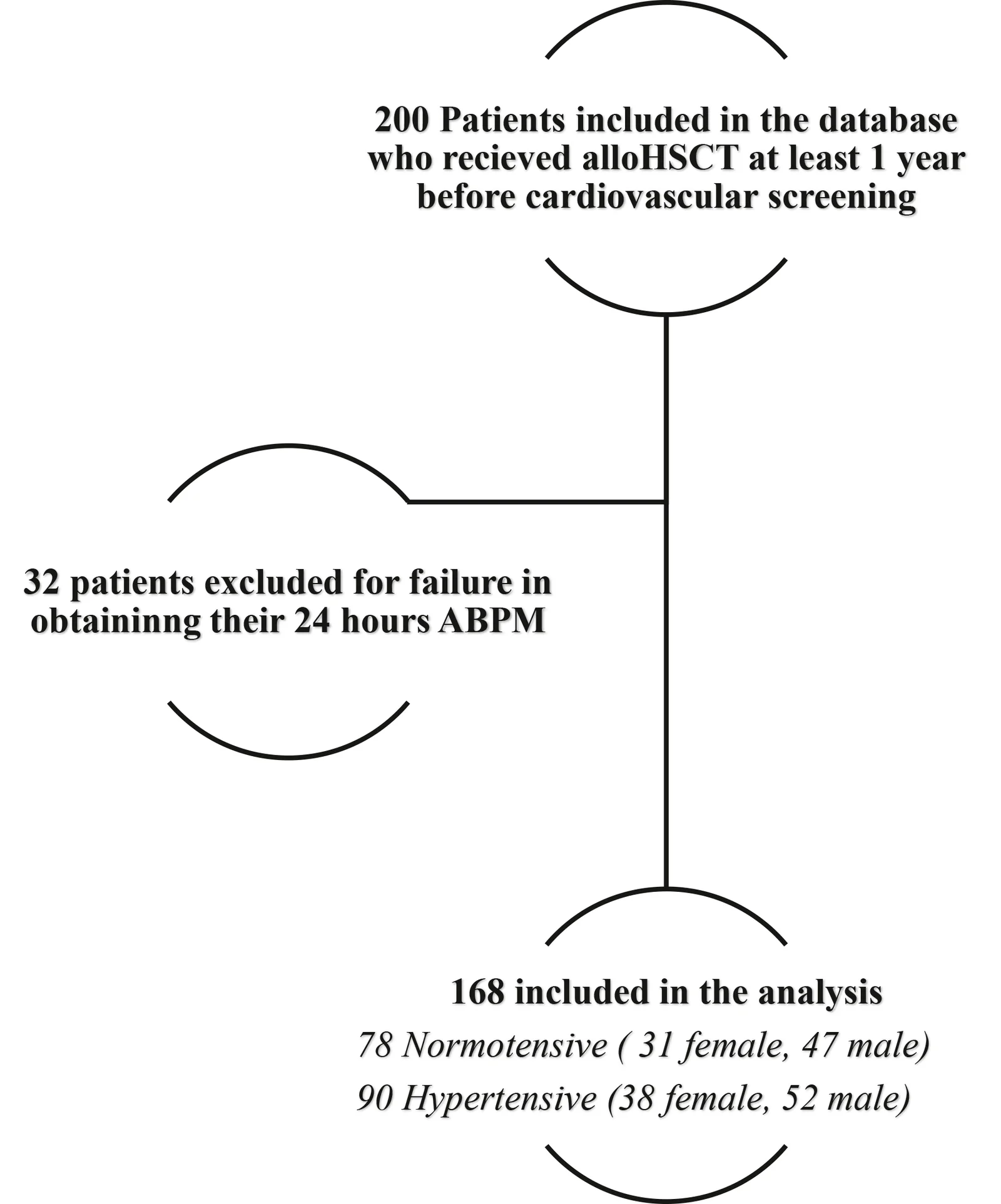
Flow Diagram of Patient Inclusion A total of 200 allogeneic hematopoietic stem cell transplantation survivors underwent cardiovascular risk assessment. Of these, 168 had valid 24-hour ambulatory blood pressure monitoring recordings and were included in the final analysis, whereas 32 were excluded because ambulatory blood pressure monitoring recordings did not meet prespecified quality criteria. The analyzed cohort was classified as normotensive (n = 78) or hypertensive (n = 90) according to ambulatory blood pressure monitoring. ABPM = ambulatory blood pressure monitoring; alloHSCT = allogeneic hematopoietic stem cell transplantation.

**Table 1:** Baseline Pretransplant and Transplant-Related Characteristics of alloHSCT Survivors According to Ambulatory Hypertension Status at Cardiovascular Screening

	Total (N = 168)	Normotensive (n = 78)	Hypertensive (n = 90)	*P* Value
Hematological profile				
Age at diagnosis (y)*,* median (IQR) [range]	49 (37.3-57.0) [14-74]	49 (27.8-57.3) [18-74]	49 (39.8-57.3) [28-75]	0.083
Years after diagnosis, median (IQR) [range]	7 (4-12) [1-35]	7 (3-11.3) [2-35]	8 (4-12) [1-21]	0.713
Years after transplant, median (IQR) [range]	5 (2-9) [1-23]	4 (2-9) [1-23]	5 (2-9) [1-21]	0.879
Sex				0.745
Female, n (%)	69 (41.1)	31 (39.7)	38 (42.2)	
Male, n (%)	99 (58.9)	47 (60.3)	52 (57.8)	
Follow-up years after alloHSCT				0.376
<5 y follow-up, n (%)	55 (32.7)	28 (35.9)	27 (30.0)	
>5-<10 y follow-up, n (%)	52 (31.0)	20 (25.6)	32 (35.6)	
>10 y follow-up, n (%)	61 (36.3)	30 (38.5)	31 (34.4)	
Hematological characteristics				0.840
AML/MDS	78 (46.4)	35 (44.9)	43 (47.8)	
ALL	25 (14.9)	14 (17.9)	11 (12.2)	
Lymphoma	27 (16.1)	14 (17.9)	13 (14.4)	
Multiple myeloma	5 (3.0)	2 (2.6)	3 (3.3)	
Others	33 (19.6)	13 (16.7)	20 (22.2)	
Transplant-related characteristics				
Disease status, n (%)				0.596
Complete remission	134 (79.8)	64 (82.1)	70 (77.8)	
Partial remission	22 (13.1)	7 (9.0)	15 (16.7)	
Stable disease	7 (4.2)	4 (5.1)	3 (3.3)	
Unknown	5 (3.0)	3 (3.8)	2 (2.2)	
Donor source, n (%)				0.316
MUD	92 (54.8)	46 (59.0)	46 (51.1)	
SIB	67 (39.9)	31 (39.7)	36 (40.0)	
HAPLO	6 (3.6)	0 (0.0)	6 (6.7)	
Cord blood	3 (1.8)	1 (1.3)	2 (2.2)	
Graft source, n (%)				0.691
BM	19 (11.3)	10 (12.8)	9 (10.0)	
PBSC	142 (84.5)	66 (84.6)	76 (84.4)	
First BM/second PBSC	2 (1.2)	1 (1.3)	1 (1.1)	
Cord	3 (1.8)	1 (1.3)	2 (2.2)	
Unknown	2 (1.2)	0 (0.0)	2 (2.2)	
Mismatch, n (%)				
HLA	17 (10.1)	4 (5.1)	13 (14.4)	0.041
ABO (blood group)	79 (47.0)	38 (48.7)	41 (45.6)	0.776
Sex	60 (35.7)	24 (30.8)	36 (40.0)	0.660
CMV	61 (36.3)	31 (39.7)	30 (33.3)	0.479
EBV	23 (13.7)	9 (11.5)	14 (15.6)	0.850
Transplant preparative regimen				0.848
TBI based	136 (81.0)	64 (82.1)	72 (80.0)	
Non-TBI based	31 (18.5)	14 (17.9)	17 (18.9)	
Type of conditioning, n (%)				0.453
NMA	61 (36.3)	31 (39.7)	30 (33.3)	
MA	76 (45.2)	36 (46.2)	40 (44.4)	
RIC	25 (14.9)	8 (10.3)	17 (18.9)	
Unknown	6 (3.6)	3 (3.8)	3 (3.3)	
GvHD (yes), n (%)				
Acute	65 (38.7)	30 (38.5)	35 (38.9)	0.874
Chronic	93 (55.4)	41 (52.6)	52 (57.8)	0.454
Use of immunosuppressants time (y)				0.335
0-1 y	82 (48.8)	41 (52.6)	41 (45.6)	
1-5 y	63 (37.5)	29 (37.2)	34 (37.8)	
5-10 y	18 (10.7)	6 (7.7)	12 (13.3)	
10-15 y	2 (1.2)	0 (0.0)	2 (2.2)	
Use of immunosuppressants at cardiovascular screening, n (%)				
Tacrolimus	19 (11.3)	5 (6.5)	14 (15.7)	0.226
Cyclosporine (CsA)	16 (9.5)	7 (9.1)	9 (10.1)	0.180
Sirolimus	2 (1.2)	1 (1.3)	1 (1.1)	0.616
Prednisone	28 (16.7)	11 (14.3)	17 (19.1)	0.612
Pretransplant cardiovascular risk factors				
Hypertension before alloHSCT (records)	8 (4.8)	2 (2.6)	6 (6.7)	0.213
Dyslipidemia before alloHSCT	8 (4.8)	4 (5.1)	4 (4.4)	0.821
Diabetes mellitus before alloHSCT	2 (1.2)	2 (2.6)	0 (0.0)	0.129
History of CVD or CV event before alloHSCT	7 (4.2)	2 (2.6)	5 (5.6)	

ALL = acute lymphoblastic leukemia; alloHSCT = allogeneic hematopoietic stem cell transplantation; AML = acute myeloid leukemia; BM = bone marrow; CMV = cytomegalovirus; CV = cardiovascular; CVD = cardiovascular disease; EBV = Epstein-Barr virus; GvHD = graft vs host disease; HAPLO = haploidentical transplant; HLA = human leukocyte antigens; MA = myeloablative; MDS = myelodysplastic syndromes; MUD = match unrelated donor; NMA = non-myeloablative; PBSC = peripheral blood stem cells; RIC = reduced-intensity conditioning; SIB = HLA-identical siblings; TBI = total-body irradiation.

Missing data: Mismatches HLA: 4, ABO: 4, Sex: 4, CMV: 5, EBV: 21, CMV reactivation: 14, Transplant preparative regimen: 1, Type of conditioning: 6, GvHD: 11, Use of immunosuppressants: 3.

**Table 2:** Blood Pressure Classification and Measurements at Cardiovascular Screening of alloHSCT Survivors (N = 168)

Age at cardiovascular screening (y), median (IQR) [range]	57 (46.3-65) [18-75]
Clinical hypertension history (y/n), n (%)	
Hypertension before alloHSCT	8 (4.8)
Clinical hypertension before ABPM screening	64 (38.1)
No prior clinical hypertension before ABPM screening	104 (61.9)
ABPM classification at screening, n (%)	
Normotensive by ABPM	78 (46.4)
Hypertensive by ABPM	90 (53.6)
Newly diagnosed hypertension by ABPM^a^	26 (15.5)
Treatment and control status, n (%)	
Use of antihypertensive medication at screening	46 (27.4)
Untreated hypertension^b^	56 (33.3)
Treated but uncontrolled hypertension^b^	34 (20.2)
Treated and controlled hypertension (normal ABPM)^c^	12 (7.1)
Office vs ABPM phenotype, n (%)^d^	
Masked hypertension	43 (25.7)
White-coat hypertension	10 (6.0)
Blood pressure measurements (mm Hg)	
Office BP, mm Hg, mean ± SD	
SBP sitting right	129 ± 18.1
DBP sitting right	81 ± 11.5
SBP sitting left	129 ± 17.6
DBP sitting left	80 ± 11.1
HR during last 2 measurements	76 ± 12.6
24-h ABPM, mm Hg, mean ± SD^e^	
24-h SBP	124 ± 13.7
24-h DBP	80 ± 10.35
24-h HR	74 ± 9.72
Daytime BP	
Daytime SBP	129 ± 13.83
Daytime DBP	81 ± 10.54
Daytime HR	77 ± 10.62
Night-time BP	
Night-time SBP	115 ± 15.95
Night-time DBP	69 ± 11.64
Night-time HR	67 ± 10.13
Arterial stiffness (PWV)	
SBP, mm Hg, PWV lying	132 ± 16.67
DBP, mm Hg, PWV lying	82 ± 11.26
HR during PWV (mean)	65 ± 10.80

ABI = ankle-brachial index; ABPM = ambulatory blood pressure monitoring; BP = blood pressure; DBP = diastolic blood pressure; HR = heart rate; PWV = pulse wave velocity; SBP = systolic blood pressure; other abbreviations as in Table 1.

*Missing data: ABI: 7, TBI: 9, PWV: 30.*

a Newly diagnosed hypertension: hypertensive ABPM without prior clinical diagnosis.

b Percentages for untreated and treated but uncontrolled hypertension are based on the total cohort; corresponding proportions among ABPM hypertensive patients (n = 90) are 62.2% and 37.8%, respectively.

c Treated and controlled hypertension: antihypertensive use with non-hypertensive ABPM.

d Phenotype classification based on patients with both office BP and ABPM available (n = 167): masked hypertension (normal office BP, hypertensive ABPM) and white-coat hypertension (elevated office BP, non-hypertensive ABPM).

e The success rate for the 24-hour ABPM is 90.5% due to the failure to obtain 16 patients’ night readings.

### Hypertension prevalence and 24-hour ABPM findings

To improve interpretability, hypertension status was classified according to prior clinical diagnosis, ABPM findings at screening, treatment and control status, and concordance between office BP and ABPM. According to medical records, the prevalence of hypertension among the 168 patients before alloHSCT was 4.8% (8 patients). At cardiovascular screening, 64 of 168 patients (38.1%) had a clinical diagnosis of hypertension prior to ABPM assessment. Based on 24-hour ABPM, the prevalence of hypertension increased to 53.6% (90 patients), whereas 78 (46.4%) were normotensive. Among patients without a prior clinical diagnosis of hypertension, 26 (15.5% of the total cohort) were newly identified as hypertensive by ABPM (Table 2). The prevalence of hypertension by ABPM was 49.1% in patients screened <5 years after alloHSCT, 61.5% in those screened 5 to 10 years after alloHSCT, and 50.8% in those screened >10 years after alloHSCT. The mean 24-hour BP was 113/69 mm Hg (SD: 8.6/6.1) in the normotensive group and 133/84 mm Hg (SD: 10.4/8.3) in the hypertensive group (Figure 2). The mean office SBP was higher (129 mm Hg) than the mean 24-hour ambulatory SBP (124 ± 13.7 mm Hg), and closely matched the daytime SBP (129 ± 13.8 mm Hg). Similarly, the mean office diastolic blood pressure (DBP) was slightly higher (81 mm Hg) than the mean 24-hour ambulatory DBP (80 ± 10.4 mm Hg), and comparable to the daytime DBP (81 ± 10.5 mm Hg). BP values obtained through other measurement methods, including night-time and PWV assessments, are presented in Table 2. Among the 90 patients with ABPM-defined hypertension, 56 of 90 (62.2%) were not receiving antihypertensive treatment and 34 of 90 (37.8%) were receiving treatment but remained uncontrolled. In addition, 12 patients (7.1% of the total cohort) were receiving antihypertensive medication and had controlled BP on ABPM. Among the 167 patients with both office BP and ABPM data available, 43 (25.7%) had masked hypertension and 10 (6.0%) had white-coat hypertension (Table 3). All hypertension parameters are summarized in Tables 2 and 3.

**Figure 2:**
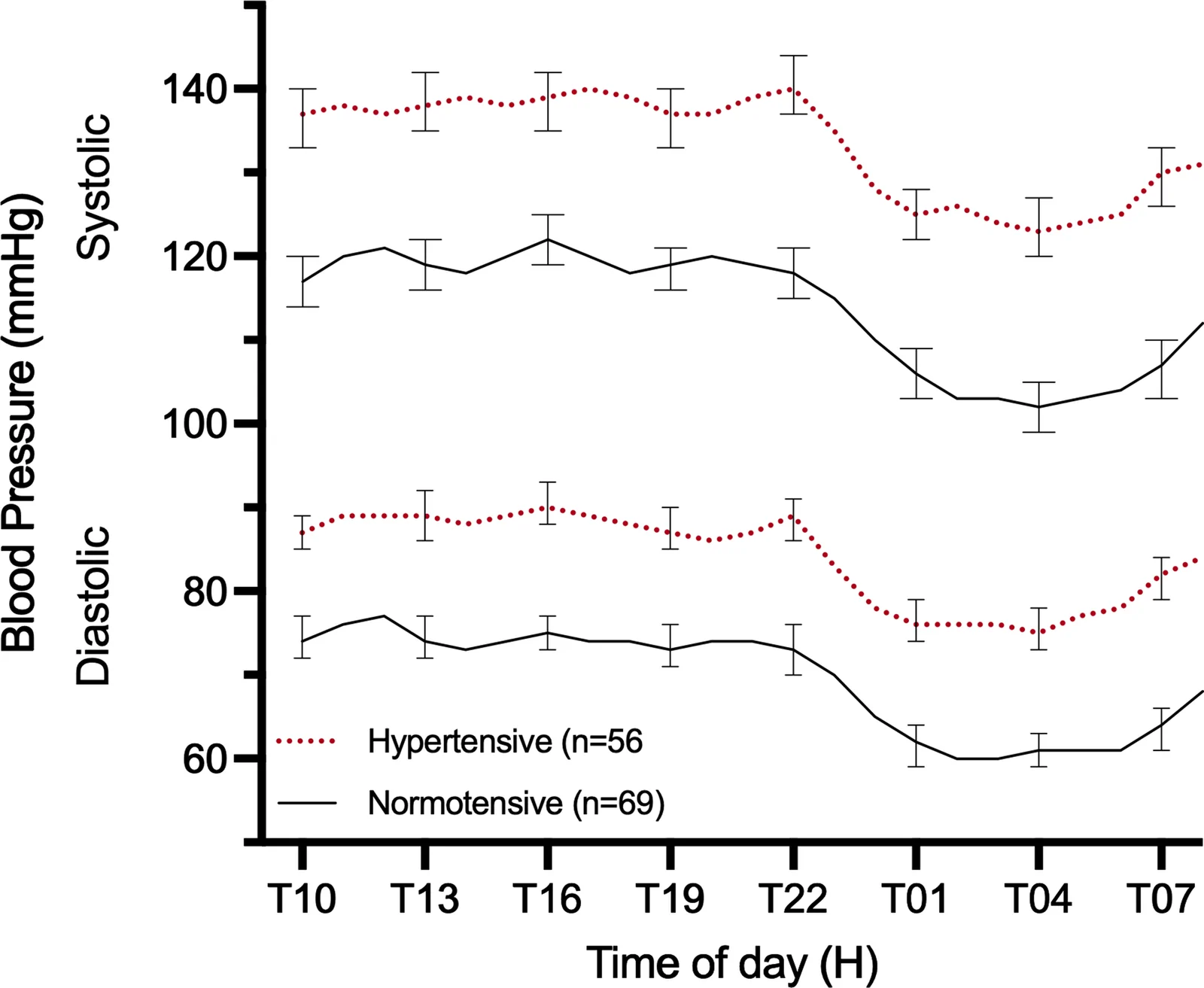
Mean Hourly 24-Hour Ambulatory Blood Pressure Monitoring in Hypertensive and Normotensive Allogeneic Hematopoietic Stem Cell Transplantation Survivors Hourly mean systolic blood pressure and diastolic blood pressure values obtained by ambulatory blood pressure monitoring are shown for hypertensive patients (dashed lines) and normotensive patients (solid lines). Values represent mean hourly measurements across the 24-hour monitoring period.

**Table 3:** Cross-Classification of Office Blood Pressure and Ambulatory Blood Pressure Monitoring at Cardiovascular Screening

	Normotensive by ABPM	Hypertensive by ABPM	Total
Normotensive by office BP	67	43	110
Hypertensive by office BP	10	47	57
Total	77	90	167^a^

Abbreviations as in Table 2.

Masked hypertension: normal office BP but hypertensive by ABPM.

White-coat hypertension: hypertensive by office BP but non-hypertensive by ABPM.

a 1 patient had missing office BP data (n = 167).

### Dipping status after alloHSCT

The dipping status of SBP and DBP was compared between normotensive and hypertensive by the ABPM groups, and the results are described in Table 4. Of the total cohort, 11 of 12 (91.7%) patients with SBP reverse dipping were hypertensive, and only one was normotensive. When comparing the SBP and the DBP dipping status between the normotensive and the hypertensive by ABPM groups, non-dipping was also significantly more common in the hypertensive group (*P* = 0.003 for the SBP dipping status and *P* < 0.001 for the DBP dipping status). Additionally, we explored the association between dipping status and subclinical organ damage. SBP non-dipping was associated with greater subclinical organ damage, reflected by lower eGFR, higher PWV, and higher IMT (Figure 3).

**Table 4:** SBP and DBP Dipping Status According to Ambulatory Hypertension Status

	Total (N = 168, 100%)	Normotensive (n = 78, 46.4%)	Hypertensive (n = 90,53.6%)	*P* Value
Dipping status (SBP), n (%)				0.003
Non-dipper	59 (35.1)	22 (28.2)	37 (41.1)	
Dipper	77 (45.8)	44 (56.4)	33 (36.7)	
Extreme dipper	14 (8.3)	9 (11.5)	5 (5.6)	
Reverse dipper	12 (7.1)	1 (1.3)	11 (12.2)	
Dipping status (DBP), n (%)				<0.001
Non-dipper	33 (19.6)	9 (11.5)	24 (26.7)	
Dipper	73 (43.5)	32 (41.0)	41 (45.6)	
Extreme dipper	49 (29.2)	35 (44.9)	14 (15.6)	
Reverse dipper	7 (4.2)	0 (0)	7 (7.8)	

Abbreviations as in Table 2.

Percentages are calculated within each column. Six patients had incomplete dipping-status data and were excluded.

**Figure 3:**
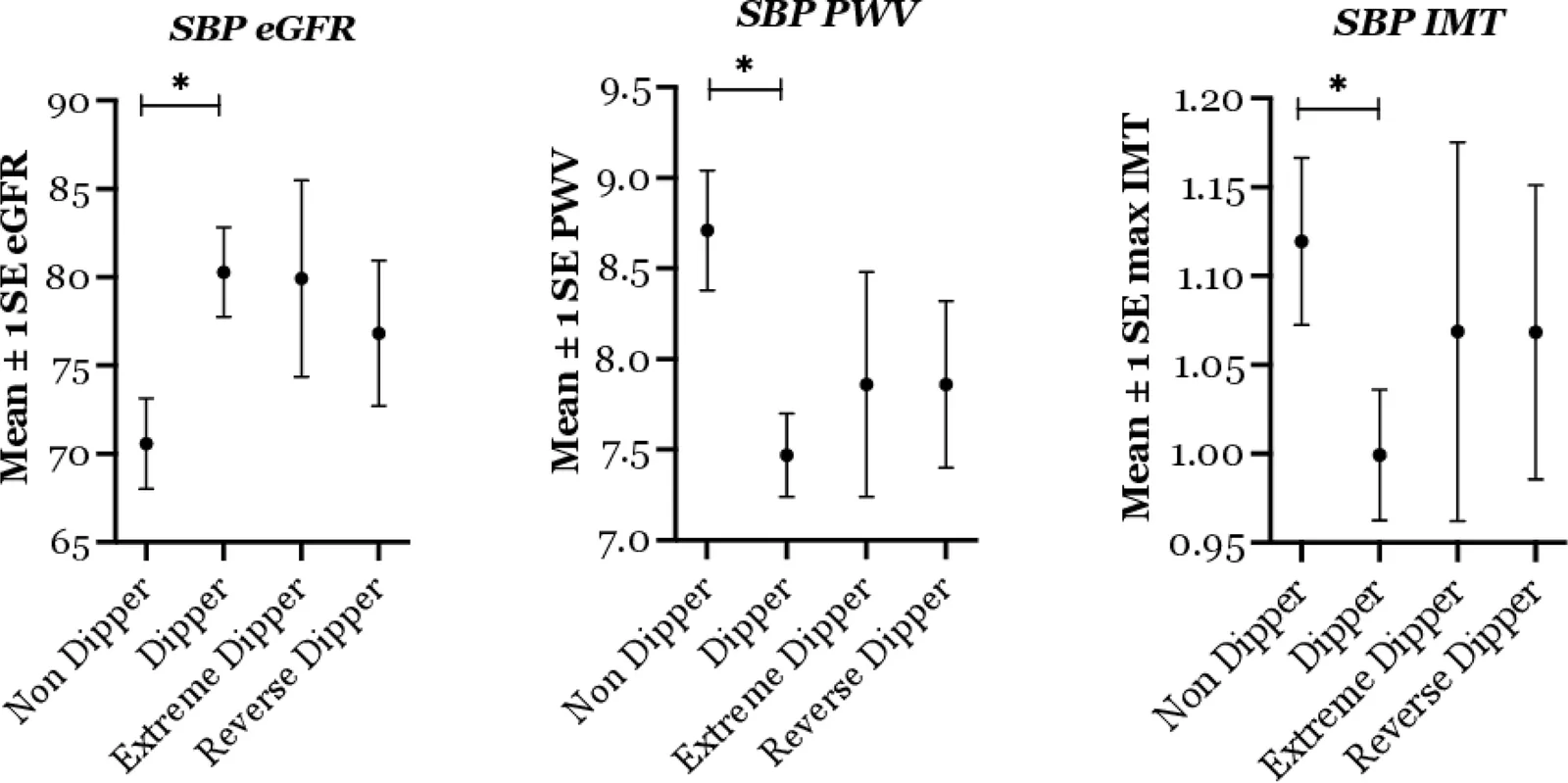
Systolic Blood Pressure Dipping Status and Subclinical Organ Damage After Allogeneic Hematopoietic Stem Cell Transplantation Geometric mean ± SE values for estimated glomerular filtration rate, pulse-wave velocity, and mean maximum carotid intima-media thickness are shown across 4 nocturnal systolic blood pressure phenotypes: non-dipper (<10% night-time systolic blood pressure fall; n = 59), dipper (10% to 20%; n = 77), extreme dipper (>20%; n = 14), and reverse dipper (night-time systolic blood pressure greater than daytime systolic blood pressure; n = 12). Overall group differences were statistically significant (*P* < 0.05). Six patients were excluded because of missing data. eGFR = estimated glomerular filtration rate; IMT = intima-media thickness; PWV = pulse-wave velocity; SBP = systolic blood pressure.

**Figure 4:**
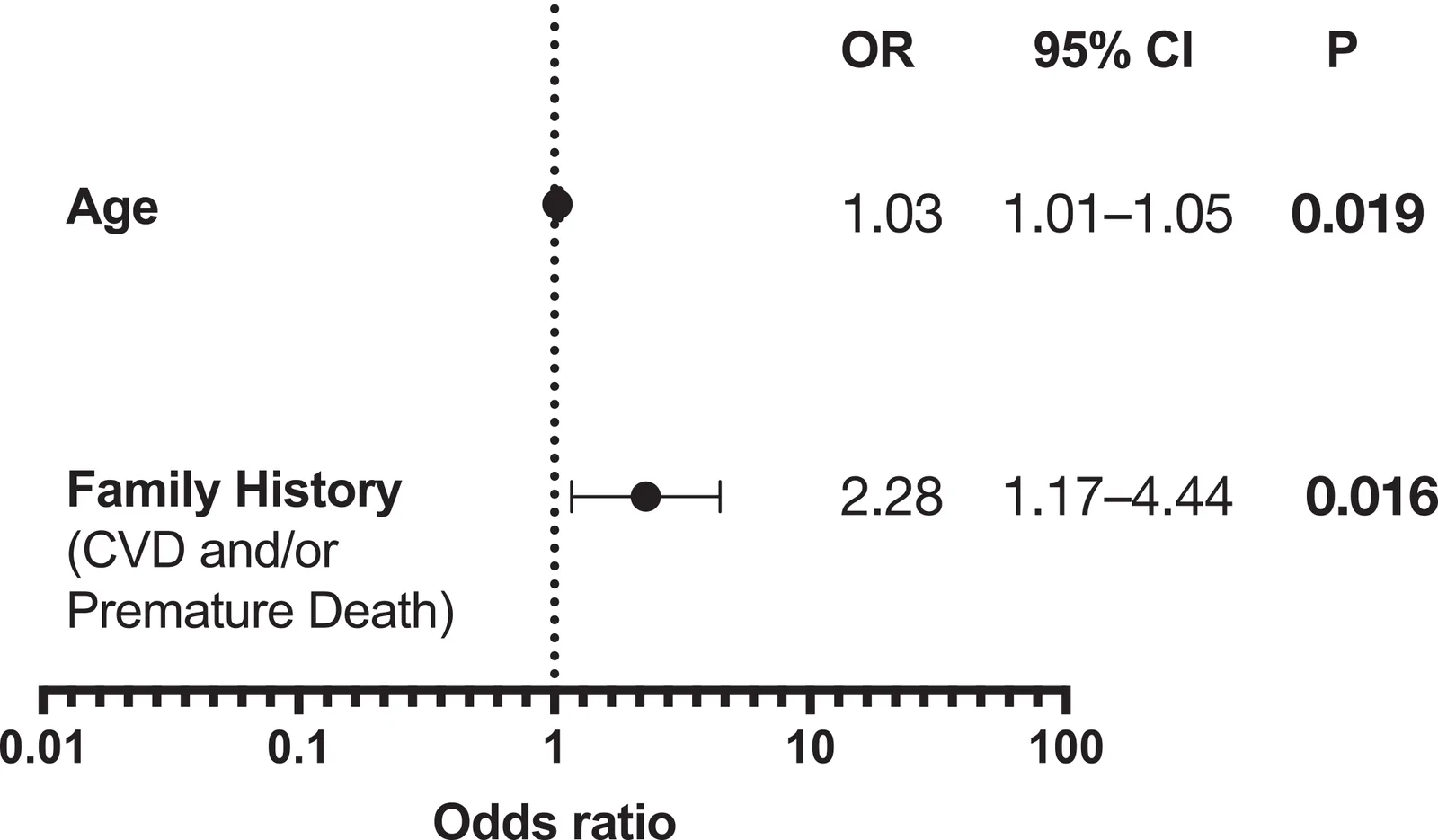
Factors Independently Associated With Ambulatory Hypertension After Allogeneic Hematopoietic Stem Cell Transplantation Forest plot showing ORs and 95% CIs for factors independently associated with ambulatory hypertension at cardiovascular screening after allogeneic hematopoietic stem cell transplantation. After backward stepwise selection of multivariable logistic regression model, only age and family history of cardiovascular disease and/or premature death remained independently associated with hypertension. Other variables entered in the model, but not retained as independent associated factors, were sex, time since transplant, acute graft-versus-host disease, chronic graft-versus-host disease, ever smoking, dyslipidemia, and use of immunosuppression for ≥1 year. CVD = cardiovascular disease.

Among hypertensive patients, 37 of 90 (41.1%) exhibited an SBP non-dipping pattern. Among these hypertensive SBP non-dippers, the mean maximum common carotid artery IMT was 1.011 ± 0.366 mm, and 8 of 37 (21.6%) had at least 2 carotid plaques. PWV data were available for 29 (32.2%) hypertensive SBP non-dippers, with a mean PWV of 9.24 ± 2.26 m/s. In addition, 13 of 37 (35.1%) hypertensive SBP non-dippers had an eGFR ≤60 mL/min/1.73 m^2^.

### Association of ambulatory hypertension with subclinical organ damage after alloHSCT

Patients with hypertension by ABPM were more likely to be male, older, have a body mass index ≥25 kg/m^2^, have eGFR ≤60 mL/min/1.73 m^2^, and have more CVRFs than normotensive by ABPM. A family history of CVD or premature death was reported in 60 (35.7%) patients and was more frequent among hypertensive by ABPM (cases 40, 44.4%) compared to normotensive by ABPM (cases 20, 25.6%) (*P* = 0.03). Markers for possible subclinical damage, such as carotid IMT and number of plaques (for atherosclerosis), creatinine and eGFR (for impaired kidney function), and PWV (for arterial stiffness) were measured and presented in Table 5. By the time of cardiovascular screening, a history of CVD or events after alloHSCT was present in 19 hypertensive patients (21.1%) and 10 nonhypertensive patients (12.8%), with no statistically significant difference between groups (*P* = 0.156) (Table 5). The mean-mean IMT and the mean-max IMT of 6 measured segments showed a significant difference between the normotensive and hypertensive by ABPM groups (*P* = 0.009 and *P* = 0.010, respectively). Mean PWV measurements using 2 different methods were also significantly different between the groups (*P* < 0.001). Finally, 34 (20.2%) patients had an eGFR ≤60 mL/min/1.73 m^2^, of whom 24 (70.6%) were hypertensive.

**Table 5:** Cardiovascular Risk Profile and Markers of Subclinical Organ Damage After alloHSCT at Cardiovascular Screening

	Normotensive 78 (46.4%)	Hypertensive 90 (53.6%)	*P* Value
Cardiovascular risk factors at screening			
Age at cardiovascular screening, median (IQR) [range]	55.5 (39.75-64) [18-74]	58 (49-67) [28-75]	0.060
BMI (kg/m^2^), median (IQR) [range]	25.62 (22.11-28.25) [17.8 -36.5]	25.70 (22.8-28.06) [15.4-44.1]	0.737
≥25 kg/m^2^, n (%)	42 (53.8)	53 (58.9)	0.511
Smoking, (y/n) n, (%)			0.080
Never	36 (46.2)	31 (34.4)	
Past	29 (37.2)	49 (54.4)	
Current	13 (16.7)	10 (11.1)	
Mean pack y	7.4 ± 12.2	8.8 ± 11.2	0.146
Family history, n (%)			0.009
CVD	20 (25.6)	39 (43.3)	0.034
Premature death	4 (5.1)	7 (7.8)	0.857
History of dyslipidemia, n (%)	49 (62.8)	63 (70.0)	0.325
History of diabetes mellitus, n (%)			0.035
Prediabetes	6 (7.7)	7 (7.8)	
Diabetes	6 (7.7)	17 (18.9)	
Pre-alloHSCT cardiovascular history			
Hypertension before alloHSCT^c^	2 (2.6)	6 (6.7)	0.213
Dyslipidemia before alloHSCT	4 (5.1)	4 (4.4)	0.821
Diabetes mellitus before alloHSCT	2 (2.6)	0 (0.0)	0.129
History of CVD/CV event before alloHSCT	2 (2.6)	5 (5.6)	
Clinical cardiovascular disease by the time of cardiovascular screening			
History of cardiovascular events after alloHSCT by the time of cardiovascular screening, n (%)	10 (12.8)	19 (21.1)	0.156
Subclinical vascular damage at screening			
Carotid intima media thickness (IMT), mm, mean ± SD			
Mean mean IMT CCA	0.70 ± 0.22	0.77 ± 0.26	0.048^a^
Mean max IMT CCA L/R	0.90 ± 0.20	0.99 ± 0.35	0.067^a^
Mean mean IMT all 6 segments	0.76 ± 0.26	0.84 ± 0.25	0.009^a^
Mean max IMT all 6 segments	1.01 ± 0.35	1.10 ± 0.33	0.010^a^
Plaque (y/n) n, (%)	17 (21.8)	16 (17.8)	0.427
Arterial stiffness (PWV), mean (SD)			
Mean PWV classic method	7.04 (1.61)	8.81 (2.14)	<0.001^a^
Mean PWV direct distance ×0.8	7.29 (1.59)	9.03 (2.27)	<0.001^a^
Ankle-brachial index (ABI)			
Right	1.13 (0.15)	1.12 (0.19)	0.650^a^
Left	1.14 (0.12)	1.13 (0.17)	0.743^a^
Toe-brachial index (TBI)			
Right	0.77 (0.18)	0.76 (0.18)	0.755^a^
Left	0.79 (0.18)	0.75 (0.18)	0.243^a^
Kidney function markers			
eGFR	80.40 (22.5)	72.30 (18.72)	0.010^a^
≤60 mL/min/1.73 m^2^, n (%) yes/no	10 (12.8)	24 (26.7)	0.026^b^

BMI = body mass index; IMT = intima-media thickness; CCA = common carotid artery; eGFR = estimated glomerular filtration rate; other abbreviations as in Tables 1 and 2.

Cardiovascular events include myocardial infarction, stroke/transient ischemic attack, peripheral arterial disease, and revascularization procedures documented by the time of cardiovascular screening.

a Values are presented as geometric means (log-transformed variables).

b Significant.

c Obtained from patients’ records.

### Factors associated with hypertension after alloHSCT

A multivariable logistic regression analysis was performed to assess the association of treatment-related and traditional cardiovascular factors with the presence of hypertension after alloHSCT. In this model, only age per year (OR: 1.03; 95% CI: 1.01-1.05; *P* = 0.02) and family history of CVD and/or premature death (OR: 2.28; 95% CI: 1.17-4.44; *P* = 0.02) reached the *P* < 0.05 threshold (Figure 4). When time since transplant (<5 years, 5-10 years, and >10 years) was added to the multivariable logistic regression model, the results remained unchanged; age and family history of CVD and/or premature death remained independently associated with hypertension, whereas time since transplant was not independently associated with hypertension. In a sensitivity analysis excluding patients with previously diagnosed hypertension before ABPM screening (n = 64), the results remained consistent. Family history of CVD and/or premature death remained independently associated with hypertension (OR: 2.42; 95% CI: 1.02-5.74; *P* = 0.045). The results of the univariate logistic regression analysis are presented in Supplemental Table 1.

## Discussion

This cross-sectional study reveals a high prevalence of hypertension among adult alloHSCT survivors when measured with 24-hour ABPM, with more than half of the cohort affected (53.6%) and a substantial proportion (25.7%) identified with masked hypertension not captured by routine office BP measurements. Hypertension in this population remains underdiagnosed (15.5%), undertreated (33.3%), and inadequately controlled at the time of cardiovascular screening. Furthermore, it was associated with subclinical vascular and renal damage, including increased carotid IMT, elevated PWV, and lower eGFR (Central Illustration).

**Central Illustration:**
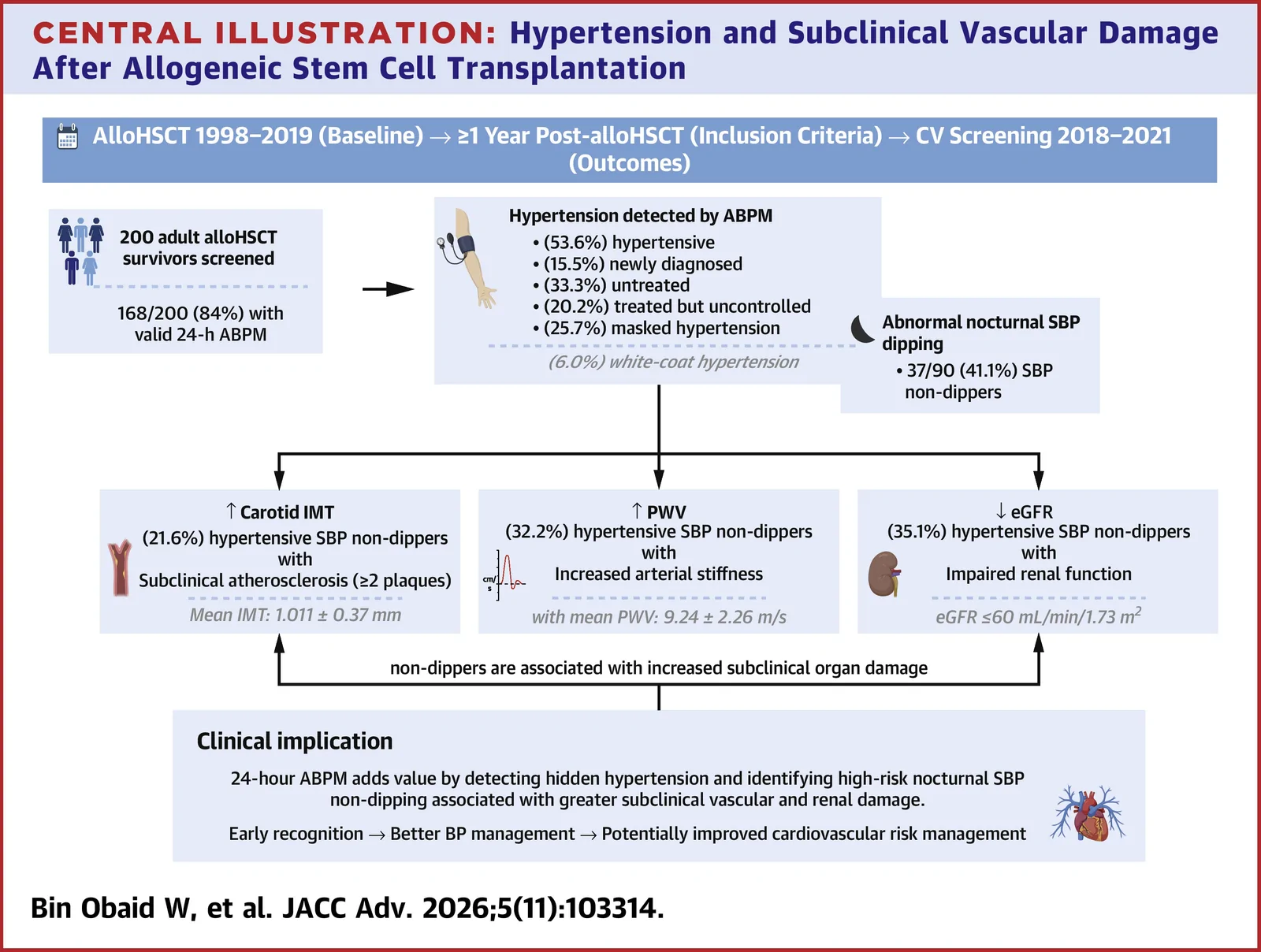
Hypertension and Subclinical Vascular Damage After Allogeneic Stem Cell Transplantation This illustration summarizes the key findings of the study. Among 200 adult allogeneic hematopoietic stem cell transplantation survivors screened, 168 completed valid 24-hour ambulatory blood pressure monitoring. Ambulatory blood pressure monitoring identified hypertension in 53.6%, including 15.5% with newly diagnosed hypertension and 25.7% with masked hypertension. Among hypertensive patients, 41.1% were systolic blood pressure non-dippers. Hypertensive systolic blood pressure non-dippers showed greater subclinical organ damage, including increased carotid intima-media thickness, higher pulse wave velocity, and impaired renal function; 21.6% had at least 2 carotid plaques and 35.1% had an estimated glomerular filtration rate ≤60 mL/min/1.73 m^2^. These findings support targeted use of 24-hour ambulatory blood pressure monitoring to detect hidden hypertension and high-risk nocturnal blood pressure patterns associated with vascular and renal damage. ∗Created using BioRender.com. CV = cardiovascular; other abbreviations as in Figures 1 and 3.

The elevated prevalence observed in our cohort aligns with prior studies among alloHSCT survivors, particularly regarding the frequency of masked hypertension and poor treatment coverage.^5^^,^^7^^,^^10^ For instance, Pao et al^19^ reported ambulatory hypertension in 72% of alloHSCT recipients (median age 48 years), underscoring the limitations of office-based screening in this population. Compared to the general population—where hypertension prevalence ranges from 30% to 45% depending on age and diagnostic thresholds—alloHSCT recipients consistently demonstrate higher rates of both diagnosed and undiagnosed hypertension.^5^^,^^10^^,^^12^ The prevalence of hypertension before alloHSCT in our cohort (4.8%) was lower than reported in other studies and should be interpreted with caution. This estimate was retrospectively derived from medical records rather than systematic pretransplant assessment and likely underestimates the true burden, particularly because ABPM was not performed and undiagnosed hypertension may have been missed. In addition, differences in age distribution, baseline patient selection, and health care context may partly explain discrepancies with larger cohorts, such as those reported in the United States. The proportion of patients in our study with unrecognized and untreated hypertension further corroborates evidence of systematic underdetection and suboptimal management in this group,^6^ supporting a broader role for ABPM in post-transplant cardiovascular surveillance, particularly in long-term survivors and in patients at increased cardiovascular risk or with suspected masked hypertension.

The use of 24-hour ABPM also revealed a high prevalence of abnormal diurnal BP patterns (particularly non-dipping and reverse-dipping), which were strongly associated with subclinical vascular damage. These patterns were especially common among hypertensive patients by ABPM, with reverse-dipping in DBP almost exclusively observed in this group and was linked to the highest burden of vascular damage, including higher IMT, increased arterial stiffness, and reduced renal function compared to dippers (Supplemental Results). This observation is consistent with findings from both general and transplant populations, where abnormal dipping patterns have been associated not only with asymptomatic subclinical organ damage—-such as atherosclerosis and increased arterial stiffness—but also with overt clinical complications, including kidney injury and CVD. These data suggest that dipping status reflects underlying mechanisms such as autonomic imbalance, inflammatory activation, and early vascular injury.^12^ Pao et al^19^ similarly reported that patients with blunted dipping and ambulatory hypertension had lower reactive hyperemia index, indicative of endothelial dysfunction, though IMT or arterial stiffness was not assessed. In pediatric alloHSCT survivors, Gurbanov et al^20^ reported higher daytime and nighttime SBP standard deviation scores in children with albuminuria or severe albuminuria, further supporting an association between elevated ambulatory BP and renal involvement after transplantation.

Several studies have reported associations between hypertension and treatment-related risk factors such as GvHD.^21^ These findings raise the question of whether patients with hypertension after alloHSCT may have different risk factors and mechanisms for developing hypertension compared to the general hypertensive population.

The regression model in our study suggested that age and family history were independently associated with the presence of hypertension. These findings were consistent in a sensitivity analysis excluding patients with previously diagnosed hypertension. In contrast, treatment-related variables (such as underlying hematological disease, remission status, donor and transplant sources, blood group mismatches, conditioning regimen, and GvHD) were not significantly associated with hypertension. Moreover, treatment-related exposures (such as total-body irradiation, conditioning intensity, and use of calcineurin inhibitors and/or steroids), were also not significantly associated with hypertension in our study, although these factors were found predictive in other adult and pediatric cohorts.^6^^,^^22^^,^^18^ Such discrepancies may reflect differences in age group, transplant type, or transplant-specific risk factors not captured in general cancer survivorship cohorts. Potential pathways include changes in hematopoiesis, shifts in gene expression of hematopoietic cells, and dysregulation of the sympathetic tone that controls BP, among other hypotheses. Therefore, CVRFs should be assessed before transplant and monitored throughout treatment, as suggested by Borchert-Mörlins et al.^5^

Additionally, a comparison between normotensive and hypertensive patients by ABPM, IMT, PWV, and eGFR revealed significant differences between the 2 groups. Specifically, hypertensive patients had higher mean IMT (0.84 ± 0.25 vs 0.76 ± 0.26 mm, *P* = 0.009), higher PWV (9.03 ± 2.27 vs 7.29 ± 1.59 m/s, *P* < 0.001), and a lower eGFR (72.3 ± 18.7 vs 80.4 ± 22.5 mL/min/1.73 m^2^, *P* = 0.010) compared to their normotensive counterparts. Moreover, the prevalence of atherosclerosis burden, impaired kidney function, and clinical CVD was higher in hypertensive patients, underscoring the association between hypertension and both subclinical and overt organ damage. Descriptive analysis of cardiovascular events documented by the time of cardiovascular screening suggested a higher proportion among hypertensive patients; however, the number of events was limited, and no causal or prognostic conclusions can be drawn. Because all vascular and BP measurements were obtained at a single screening visit, our data do not allow conclusions regarding the prognostic value of these subclinical markers for subsequent cardiovascular events in alloHSCT survivors.

To our knowledge, this is among the first adult alloHSCT studies to combine 24-hour ABPM, dipping status, and three validated measures of subclinical organ damage (IMT, PWV, and eGFR) in alloHSCT survivors. Moreover, the clustering of masked hypertension, non-dipping, and reverse dipping in this population underscores the inadequacy of office BP in detecting true cardiovascular risk and supports a broader role of ABPM and vascular assessment in long-term alloHSCT follow-up. Although 24-hour ABPM is considered the reference standard for diagnosing hypertension, routine use in all alloHSCT survivors may not be feasible because of cost, availability, patient burden, and the need for specialized interpretation. Therefore, our findings do not support universal ABPM screening but rather suggest that ABPM may be particularly useful in selected higher-risk patients, including long-term survivors, those with multiple CVRFs, prolonged immunosuppressive therapy, impaired renal function, or suspected masked hypertension despite normal office BP measurements. Future prospective studies should determine which alloHSCT survivors derive the greatest benefit from ABPM and evaluate the cost-effectiveness and clinical impact of targeted ABPM-guided cardiovascular screening and management.

### Strengths and limitations

To the best of our knowledge, this is among the first adult alloHSCT studies to report an association between abnormal diurnal BP profiles, particularly non-dipping, and structural (carotid IMT) and functional (PWV) vascular damage. Unlike previous work limited to endothelial surrogates, our findings show a cross-sectional association between dipping status and subclinical vascular injury in long-term transplant survivors.

A limitation of this study is its cross-sectional design, with cardiovascular data collected at a single screening visit. Because both BP and vascular markers were assessed at one time point, no conclusions can be drawn regarding causality or the prognostic value of these findings for subsequent cardiovascular events. In addition, pretransplant hypertension was retrospectively derived from medical records and may have been underestimated because ABPM was not performed and undiagnosed hypertension may have been missed. Detailed data on GvHD severity and activity at the time of cardiovascular screening, as well as cumulative exposure to specific immunosuppressive agents, were not available for all patients. Although duration of immunosuppressive therapy may partly reflect GvHD burden, this limited granularity may have reduced our ability to fully assess the contribution of GvHD-related factors and treatment exposures to hypertension and vascular dysfunction. Furthermore, this was a single-center study conducted at one large tertiary referral center only, and no external control group was available for comparison. The relatively modest sample size in relation to the number of candidate variables initially considered may also have increased the risk of model overfitting, unstable coefficient estimates, and limited statistical power. Therefore, the multivariable regression findings should be interpreted with appropriate caution and require confirmation in larger prospective cohorts. Nevertheless, these limitations do not compromise our primary aim of the study, namely to assess the prevalence of hypertension, factors associated with hypertension, and its cross-sectional association with subclinical organ damage in alloHSCT survivors. Future prospective longitudinal studies with larger cohorts are needed to assess the cumulative impact of hypertension, other CVRFs, and subclinical organ damage on accelerated cardiovascular aging after alloHSCT. Finally, fundamental research is warranted to elucidate the mechanisms behind the increased prevalence of hypertension in alloHSCT survivors.

## Conclusion

Hypertension in long-term survivors after alloHSCT is highly prevalent, underdiagnosed, undertreated, and associated with subclinical organ damage. This population would benefit from regular cardiovascular screening and long-term monitoring after transplant to reduce CV complications. Twenty-four-hour ABPM is a useful noninvasive tool that not only improves the diagnosis of hypertension but also provides additional value in identifying asymptomatic subclinical and overt organ damage.

Perspectives **COMPETENCY IN MEDICAL KNOWLEDGE:** Adult alloHSCT survivors have a high prevalence of previously unrecognized hypertension detectable with 24-hour ABPM. This is associated with subclinical organ damage and highlights the need for structured cardiovascular risk assessment, optimization of antihypertensive treatment, and long-term follow-up, since not all patients with previously diagnosed hypertension achieved adequate BP control on treatment.

**TRANSLATIONAL OUTLOOK:** Future prospective longitudinal studies should determine whether aggressive BP control and targeted therapies can reverse or slow the progression of subclinical organ damage, thereby reducing cardiovascular morbidity in the expanding population of alloHSCT survivors.

## Funding support and author disclosures

This research was supported by institutional support from the University Medical Center Groningen (Groningen, the Netherlands) and by a scholarship from the Saudi Ministry of Education (Riyadh, Saudi Arabia). The authors have reported that they have no relationships relevant to the contents of this paper to disclose.
