# Supplementary material for: Hypertension and Subclinical Vascular Damage After Allogeneic Hematopoietic Stem Cell Transplantation
Source: JACC Adv. 2026 Sep 22;5(11):103314. doi: 10.1016/j.jacadv.2026.103314 (PMC13721015; doi:10.1016/j.jacadv.2026.103314)
Supplement: Supplementary file 1 [file mmc1.docx]

**Supplemental Table 1. Univariate logistic regression analysis of factors associated with hypertension after alloHSCT**

| **Parameter** | **Coefficient** | **Odds Ratio** | **95% CI (Lower – Upper)** | **p-value** |
| --- | --- | --- | --- | --- |
| **Age** | 0.029 | 1.029 | 1.006 – 1.053 | 0.014** |
| **Sex** | -0.103 | 0.903 | 0.487 – 1.673 | 0.745 |
| **Years after transplant** | -0.012 | 0.988 | 0.930–1.050 | 0.705 |
| **Follow-up duration (<5 years reference)** |  |  |  | 0.378 |
| **5–10 years** | 0.526 | 1.66 | 0.77–3.58 | 0.197 |
| **>10 years** | 0.069 | 1.07 | 0.52–2.22 | 0.852 |
| **Conditioning regimen** | -0.277 | 0.758 | 0.403 – 1.424 | 0.389 |
| **TBI dose > 8 Gy** | -0.577 | 0.561 | 0.256 – 1.232 | 0.150 |
| **HLA mismatch** | 1.125 | 3.079 | 0.959 – 9.882 | 0.059* |
| **Use of immunosuppressants (≥1 year)** | 0.316 | 1.371 | 0.742 – 2.535 | 0.313 |
| **Acute GvHD** | 0.051 | 1.052 | 0.561 – 1.975 | 0.874 |
| **Chronic GvHD** | 0.238 | 1.268 | 0.681 – 2.363 | 0.454 |
| **A + C GvHD** | -0.021 | 0.979 | 0.498 – 1.923 | 0.951 |
| **History of Diabetes mellitus after alloHSCT** | 1.028 | 2.795 | 1.043 – 7.491 | 0.041** |
| **History of Dyslipidemia after alloHSCT** | 0.323 | 1.381 | 0.726 – 2.628 | 0.326 |
| **History of CVD or CV event** | 0.599 | 1.820 | 0.790 – 4.193 | 0.160 |
| **Family history of CVD or premature death** | 0.885 | 2.423 | 1.276 – 4.600 | 0.007** |
| **History of Smoking (ever)** | 0.441 | 1.554 | 0.836 – 2.888 | 0.164 |
| **BMI > 30** | -0.437 | 0.646 | 0.282 – 1.480 | 0.302 |
| **eGFR < 60** | 0.905 | 2.473 | 1.098 – 5.568 | 0.029** |

* p ≤ 0.10

**p ≤ 0.05
